# Seed-mediated Electrochemically Developed Au Nanostructures with Boosted Sensing Properties: An Implication for Non-enzymatic Glucose Detection

**DOI:** 10.1038/s41598-020-64082-5

**Published:** 2020-04-29

**Authors:** Hossein Siampour, Sara Abbasian, Ahmad Moshaii, Kobra Omidfar, Mosslim Sedghi, Hossein Naderi-Manesh

**Affiliations:** 1 Department of Physics, Tarbiat Modares University, P.O Box, 14115-175 Tehran, Iran; 2 School of Physics, Institute for Research in Fundamental Sciences (IPM), P.O. Box, 19395-5531 Tehran, Iran; 3 Biosensor Research Center, Endocrinology and Metabolism Molecular-Cellular Sciences Institute, Tehran University of Medical Science, Tehran, Iran; 4 Department of Nanobiotechnology/Biophysics, Faculty of Biological Sciences, Tarbiat Modares University, Tehran, 14115-154 Iran

**Keywords:** Other nanotechnology, Materials for devices, Nanoscale materials

## Abstract

A new approach has been developed to improve sensing performances of electrochemically grown Au nanostructures (AuNSs) based on the pre-seeding of the electrode. The pre-seeding modification is simply carried out by vacuum thermal deposition of 5 nm thin film of Au on the substrate followed by thermal annealing at 500 °C. The electrochemical growth of AuNSs on the pre-seeded substrates leads to impressive electrochemical responses of the electrode owing to the seeding modification. The dependence of the morphology and the electrochemical properties of the AuNSs on various deposition potentials and times have been investigated. For the positive potentials, the pre-seeding leads to the growth of porous and hole-possess networks of AuNSs on the surface. For the negative potentials, AuNSs with carved stone ball shapes are produced. The superior electrode was achieved from AuNSs developed at 0.1 V for 900 s with pre-seeding modification. The sensing properties of the superior electrode toward glucose detection show a high sensitivity of 184.9 µA mM^−1^ cm^−2^, with a remarkable detection limit of 0.32 µM and a wide range of linearity. The excellent selectivity and reproducibility of the sensors propose the current approach as a large-scale production route for non-enzymatic glucose detection.

## Introduction

During past decades, electrochemical sensing has attracted many attentions due to their considerable advantages such as high sensitivity, low cost, fast response time, wide linear range and portability and widespread applications^1–9^. Up to now various types of nanomaterial such as carbon nanotubes^10^, graphene^11,12^, noble metals^13,14^, metal oxides^15^, hybrid^16^ and composite nanostructures^17,18^ have been developed for electrochemical sensing. Among different nanomaterials, gold nanostructures (AuNSs) have attracted significant interests because of their remarkable electrical and catalytic properties, easy functionalization and excellent biocompatibility.

Since the electron transfer and catalytic activity of AuNSs strongly depend on their morphology and their size, extensive researches have been proposed in literatures to develop optimized AuNSs for sensing applications^19,20^. The notable approaches between them are based on the electrodeposition^19^, direct electrostatic self-assembly^21^, self-assembly with polymer^22–24^ and seed-mediated growth^25–28^. In the cases of self-assembly and seed-mediated strategies, the electron transfer and catalytic activity of AuNSs are hindered due to the existence of surfactant and linker molecules surrounding the nanostructures. The electrodeposition is the most reliable approach for shape and size controlled construction of AuNSs due to the possibility of controlling the nucleation and the growth processes. However, in this method the well control of morphology is provided by using template, surfactant or pre-treatment of the substrate that limit the applicability of the method^20,29,30^.

On the other hand, fabrication of nanoparticles by physical vapor deposition (PVD) provides a cost-effective and excellent reproducible method to produce nanoparticles directly on the substrate^31,32^. However, percolation of nanoparticles to form a thin film by increasing deposition thickness and limitation in shape diversity have restricted widespread application of PVD in biosensing^33^. Therefore direct construction of pure AuNSs with efficient substrate coverage without using templates, surfactant and stabilizer is a considerable challenge.

In the current work, a two-step approach is introduced to enhance the electrochemical performance of AuNSs deposited on an FTO substrate. In this regards, physical vapor deposition and annealing treatment have been utilized to modify an FTO substrate by seeding Au nanoparticles (AuNPs). The pure AuNSs have been deposited on the pre-seeded electrode by electrodeposition in very low concentrations of HAuCl_4_. The electrodeposition has been carried out at different potentials and times to investigate the effect of deposition parameters on the morphology and electrochemical properties of AuNSs. The results show that in addition to applying potential, pre-seeding of the substrate strongly influence the morphology and electrochemical properties of AuNSs. In addtition, the seeding modification leads to selective deposition and growth of AuNSs in the electrodeposition. Based on electrochemical results, the AuNSs deposited at 0.1 V for 900 s on the pre-seeded substrate has been selected as the optimum electrode for supplementary non-enzymatic detection of glucose. Due to biocompatibility of gold, the proposed Au-based electrodes in this work can be used for detection of many biomarkers in the forms of immunosensors and aptasensors. While the electrodes made by other transition metals in the non-enzymatic glucose detections cannot be used for various electrochemical immunosensing/aptasensing applications.

## Results

### Morphology characterization of AuNSs

**Figure 1:**
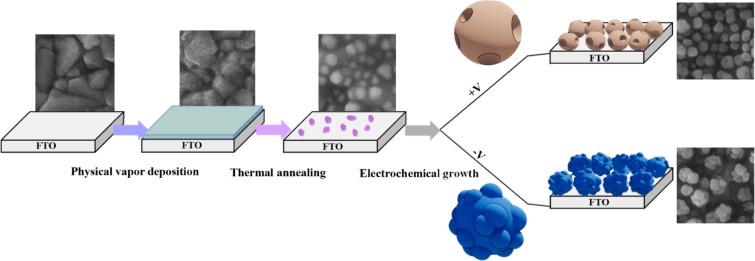
Schematic representation of the fabrication procedure of AuNSs by combining the physical vapor deposition, the thermal annealing, and the electrochemical growth on the FTO substrate.

Fabrication procedure of AuNSs on the pre-seeded FTO is shown schematically in Fig. 1. To form seeding Au nanoparticles, a 5 nm Au thin film has been thermally deposited on FTO followed with the thermal annealing treatment at 500 °C. Figure S1 (in Supporting Information) shows the SEM images of the FTO substrate, the 5 nm Au thin film and the pre-seeded substrate, respectively. The Au thin film is physisorbed on the surface with a weak adhesion. By applying the annealing procedure, the Au nanoparticles are first formed on the surface and then these nanoparticles are gradually embedded in the glass of FTO because the annealing temperature is near the transition temperature of glass^34,35^. Such embedded Au nanoparticles in the glass are highly stable and act as seeding nanoparticles for the growth of AuNSs^34,36,37^.

After that, AuNSs have been electrodeposited on the pre-seeded substrate. By adjusting electrodeposition parameters, different sizes and various morphologies of the nanostructures can be produced^19,38–40^. Generally, the growth process of AuNSs are influenced by the deposition potential^19,41^, deposition time^42,43^, concentration and pH of HAuCl_4_ electrolyte^19,43^ and the surfactants used^44–48^. Here, an aqueous solution of HAuCl_4_ (0.5 mM) with pH of 3.2 without using any surfactant was used. The pH of the aqueous solution of 0.5 mM HAuCl_4_ was measured as 3.2. Therefore, the kinetics of the growth are controlled by the deposition potential and the deposition time. In order to study the effect of seeding modification, the AuNSs have electrochemically been deposited on the bare and the pre-seeded FTO substrates under the same conditions.

**Figure 2:**
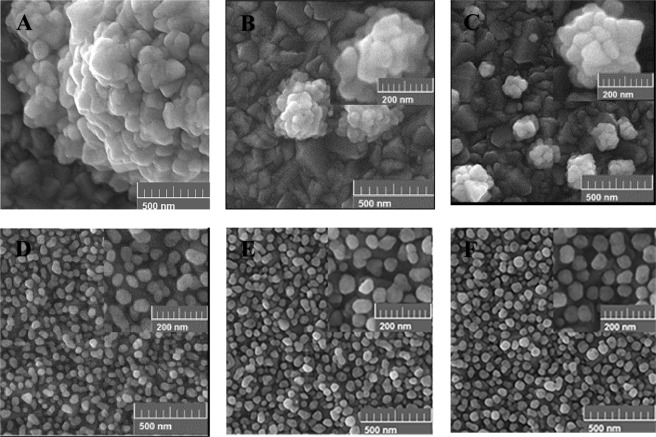
Effect of the positive electrodeposition potential on the morphology of the AuNSs grown on the bare (**A–C**) and the pre-seeded FTO electrodes (**D**–**F**) for the deposition time of 900 s at different applying potentials of; (**A,D**) 0.5 V, (**B,E**) 0.3 V, and (**C,F**) 0.1 V.

**Figure 3:**
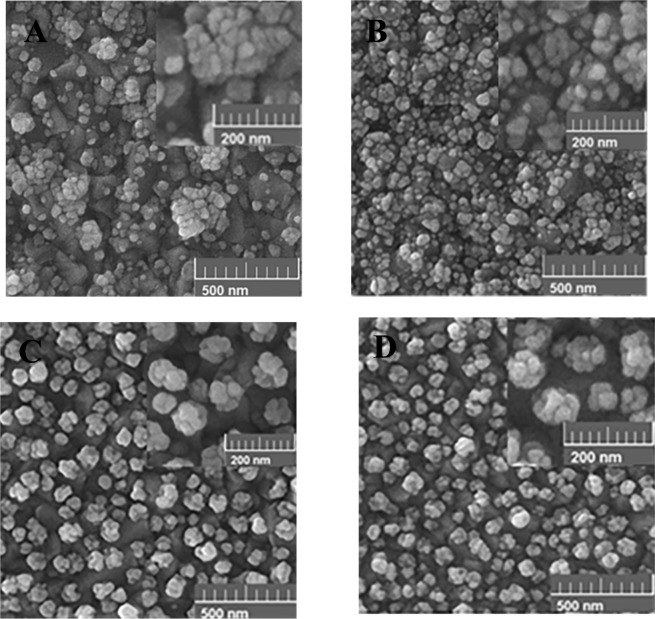
Effect of various negative potentials on the morphology of the AuNSs grown on the bare (**A,B**) and the pre-seeded FTO electrodes (**C,D**) for the 900 s time interval including: (**A,C**) -0.1 V and (**B,D**) −0.3 V.

Generally, the morphology of AuNSs is strongly affected by the deposition potential. Different potentials in the range of −0.3 V to +0.5 V were applied. Figures 2 and 3 show the SEM images of the grown AuNSs on the bare and the pre-seeded FTO substrates at different positive (Fig. 2) and negative (Fig. 3) potentials, for time interval of 900 s. Comparing the images of the bare and the pre-seeded substrates at each deposition potential indicates considerable differences in the growth pattern, distribution, and coverage of the nanostructures. As shown in Fig. 2(A–C), for the bare FTO, sparse flower-like AuNSs are formed randomly. Also, the density of AuNSs increases gradually with the deposition potential from 0.5 V to 0.1 V, due to more nucleation on the surface. Figure 2(D–F) shows the growth behavior of AuNSs when the electrode is modified by the seeds of AuNPs. The pre-seeding of the substrate forms a dense array of semi-spherical AuNPs which is completely distinguishable from the bare electrode. By decreasing deposition potential from 0.5 V to 0.1 V, AuNPs become more roundish and uniform. In addition, at deposition potentials of 0.3 V and 0.1 V some tiny holes are observed on the AuNSs. The tiny holes are more observable for the lower potentials. It seems, in the case of the pre-seeded substrate, the AuNSs selectively grow on the seed AuNPs on the surface.

The cause of formation of the holes on the AuNSs can be related to two factors of the crystallographic surfaces of the seeded Au-NPs^49^ and the growth mechanism in the electrodeposition process^50^. Different crystallographic surfaces of the seeded AuNPs may have different growth activities under the same electrochemical conditions. Therefore, the growth rate can be different for various crystallographic surfaces. On the other hand, the mechanism of growth depends on two kinetic factors including chemical diffusion of the gold ions and the subsequent reduction reactions on the electrode surface. Accordingly, by controlling the ratio of the reaction to the diffusion for the gold ions, different shapes of the AuNSs can be produced. In the case of positive deposition potentials, the low rate of the reactions on the surface leads to a reaction-limited growth process. In this case, the seeded AuNPs are surrounded by sufficient ions. Consequently, under the positive potentials, the AuNSs grow with spherical shape in addition to some holes on their surfaces.

Figure 3 shows the effect of various negative potentials on the morphology of AuNSs on the bare and the pre-seeded gold electrodes. The results show that the morphologies of AuNSs are considerably different for the bare and the pre-seeded electrodes under applying negative potentials. For the bare FTO substrate, in comparison to the positive potentials in Fig. 2, the flower-like shapes change to some irregular aggregated AuNPs shown in Fig. 3A,B. Moreover, with applying greater negative potentials, the density of AuNSs increases on the surface due to more fabricated nuclei of AuNPs. For the pre-seeded electrode (Fig. 3C,D), the Au morphology is similar to carved stone balls. Of course, no considerable difference is identified in the morphology of AuNSs with decreasing the potential from −0.1 V to −0.3 V. Similar to the positive potentials, the surface morphology of the pre-seeded electrode is clearly different from the bare one. In addition, the existence of almost regular growth of AuNSs on the pre-seeded substrate indicates the selective growth of AuNSs on the seeding AuNPs on the surface. In this case, the mechanism of the growth is mostly diffusion mode and a gradient of gold ions’ concentration is produced around the seeding AuNPs^50^.

**Figure 4:**
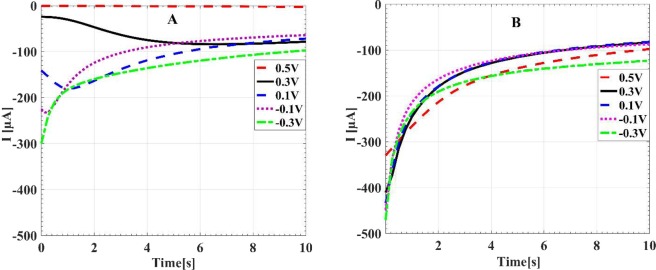
Chronoamperometry curves of electrodeposition of gold on the bare (**A**) and the pre-seeded FTO electrode (**B**), for different deposition potentials.

To have a better insight on the selective deposition and growth of AuNSs on the pre-seeded electrode, chronoamperometry technique was utilized. It is well known that the chronoamperometry technique gives details of the transient current^38,41,51^. Figure 4A shows the transient current-time (I-t) curve during the electrodeposition on the bare electrode. For more negative potentials, due to the fast formation of the nucleus on the surface of the electrode, higher currents are observed in the earliest times of the characterizations. We see that for the potential 0.5 V, a tiny current less than 100 µA is measured. For the deposition potentials of 0.3 V and 0.1 V, the current is below 200 µA. However, for the potentials of −0.1 and −0.3 V, the maximum current is between 200–300 µA. In the Fig. 4B, we see that with seeding modification of the surface, the initial currents of the samples are considerably greater than the similar cases without pre-seeding. The initial current for all voltages are more than 300 µA, which is even greater than the peak current between the samples without pre-seeding. The time evolution of the current in the pre-seeded samples can be described by the inverse square root of the time which is known as the Cottrell equation^51^.

1 $$I(t)=nFA{C}_{0}{\left(\frac{D}{\pi t}\right)}^{\frac{1}{2}}$$

where, I(t) is the current, *n* is the number of electrons involving in the reduction reaction, F is the Faraday constant, A is the surface area, D is the diffusion coefficient and C_0_ is the initial concentration of electroactive species. With changing the electrodeposition potential, the density of electrons involved in the deposition changes and therefore we see different inverse-square curves for different potentials.

A distinct difference between the curves of the pre-seeded and the bare samples is the lack of the negative peak of the current in the pre-seeded sample as shown in Fig. S2. Such peak generally relates to nucleation of tiny AuNPs on the bare samples^38,51,52^. This causes that the current increases in the early stages of the deposition for the bare sample. However for later times, the covering of the surface by the AuNSs leads to decrement of the current and convolution of these two phenomena leads to a negative peak in the current for the bare surfaces. However, for the pre-seeded substrate, the nucleation process cannot take place due to the existence of the seeding AuNPs and consequently the only covering stage, which is proportional to the inverse square root of the time, is seen for the current. In the pre-seeded samples, the electrodeposition of gold mainly occurs onto the seeded Au places rather than on the defect places of the substrate^53,54^.

**Figure 5:**
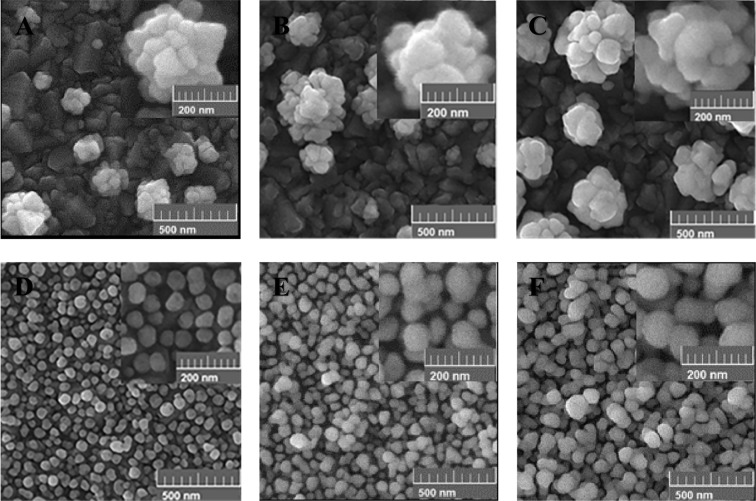
Effect of the deposition time on the morphology of the AuNSs grown on the bare (**A–C**) and the pre-seeded FTO electrodes (**D–F**) at 0.1 V for different deposition times of 900 s (**A,D**), 1800 s (**B,E**) and 3600 s (**C,F**).

The deposition time is another parameter affecting the morphology and the electrochemical properties of the fabricated AuNSs. Figure 5 shows the morphological evolution of AuNSs deposited at 0.1 V on the bare and the pre-seed electrodes with different deposition times. The selection of the potential of 0.1 V, is based on the best current response of this potential between all examined positive and negative deposition potentials. In the case of the bare electrode (Fig. 5A–C), as the deposition time increases from 900 s to 3600 s, the size of flower-like AuNSs increases due to the formation of more branches on the existing nuclei, without noticeable increment in the density of the nanostructures. For the pre-seeded electrodes (Fig. 5D–F), longer deposition times of 1800 s and 3600 s lead to formation of a porous network of nanostructures, which gradually cover all of the substrate. It should be mentioned that the observed holes on the AuNSs at 900 s disappear for longer deposition times of 1800 s and 3600 s.

The further characterizations of various samples have been carried out by XRD and AFM and the results have been shown in the supporting information (Figs. S3 and S4). These characterizations clearly support the formation of pre-seeding AuNPs on the FTO substrate and subsequent growth of AuNSs by the electrodeposition.

### Electrochemical performance of the AuNSs

In order to study the electrochemical behaviors of different electrodes before growth of AuNSs, the cyclic voltammetry (CV) and electrochemical impedance spectroscopy (EIS) measurements have been carried out for the three electrodes of the bare FTO (BFTO), 5 nm Au on FTO and the the pre-seeded FTO (SFTO) and the results are shown in Fig. S5. The CV curves have been obtained at the potential window of 0–0.6 V in the solution KCl (0.1 M) containing redox couple of [Fe (CN)_6_]^3-/4-^ (2.5 mM), at the scan rate of 50 mV s^−1^. In addition, the EIS results at the frequency range of 1 MHz to 0.1 Hz were acquired.

The cyclic voltammograms of the three electrodes are shown in Fig. S5A. It is seen that after the modification of the BFTO by the ultrathin Au film, a pair of redox peaks appear in the cyclic curves, which is accompanied with a remarkable increase in both anodic and cathodic peaks. It is also seen that the voltage difference between the oxidation and reduction peaks (identified as ΔE_P_) decreases with the Au modification of the surface. Thermal processing of the 5 nm Au film leads to more increasing in the intensity of current peaks in addition to more decreasing ΔE_P_. Therefore, an improved sensing behavior from the modified electrode is expected based on the shown electrochemical characterizations.

In addition to the CV study mentioned here, EIS analysis can provide further information since the diameter in the Nyquist plots equals the electron-transfer resistance (described as R_et_). Figure S5B shows the Nyquist plots of the BFTO, 5 nm Au/FTO and the SFTO. For the BFTO, a large R_et_ is observed indicating a great resistance against charge transfer at the bare electrode surface. After deposition of the ultrathin Au film, a notable decrease in R_et_ occurs. A further decrease in R_et_ is observed after thermal treatment of the ultrathin Au film, which mostly relates to increment of active surface area of the electrode due to formation of seeds of AuNPs.

**Figure 6:**
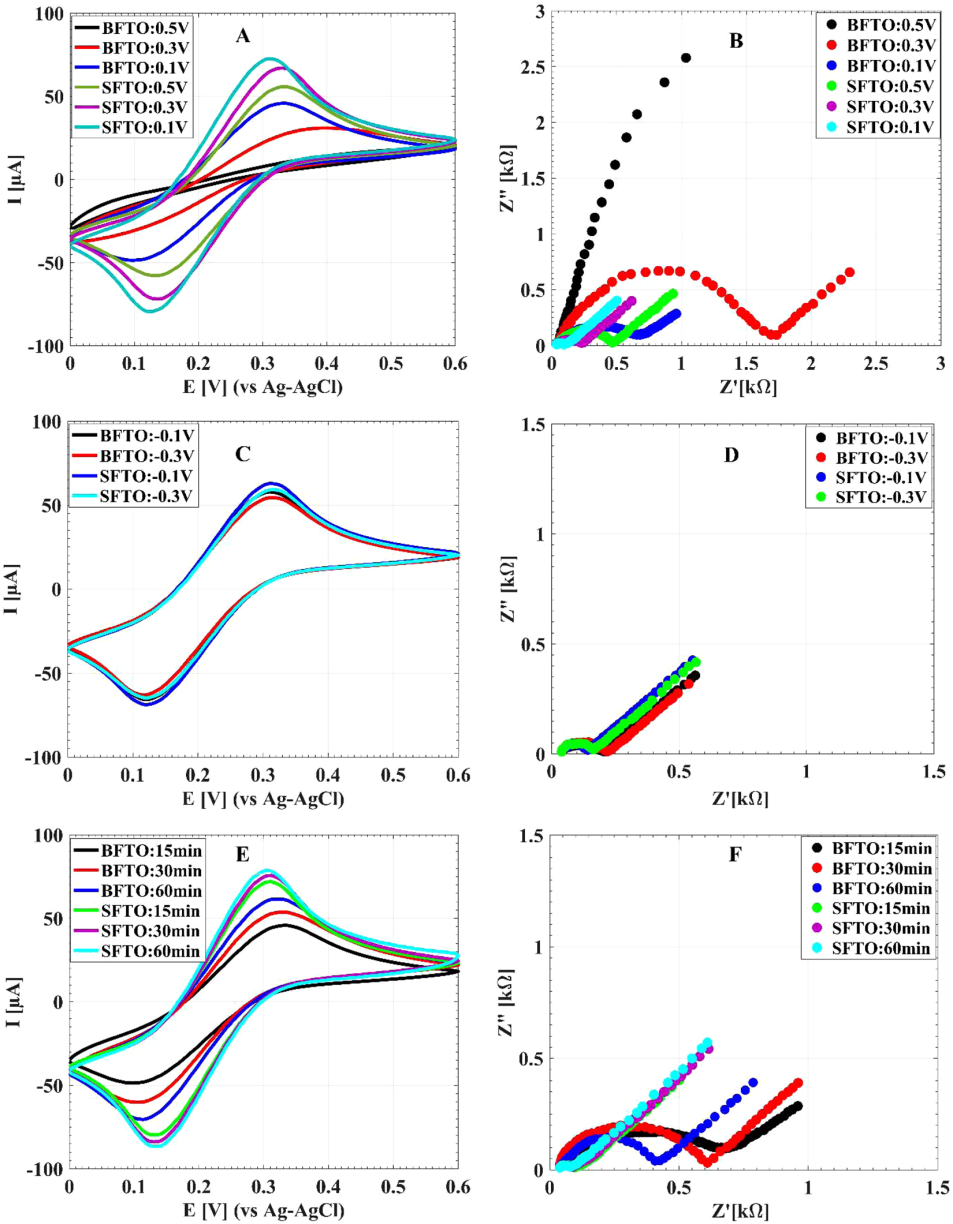
The CV and EIS responses of the BFTO and the SFTO after electrodeposition of AuNSs. Graphes A and B display the CV and EIS responses of AuNSs grown during 900 s with different positive deposition potentials. Graphes C and D display the CV and EIS results for the 900 s growth of AuNSs with various negative deposition potentials. Graphes E and F display the CV and EIS results for the optimum deposition voltage of 0.1 V with various deposition times.

The electrochemical performances of the electrodeposited AuNSs on the SFTO in comparison with those of the BFTO for different deposition potentials and times are shown in Fig. 6. The CV and EIS curves for different positive potentials with fixed deposition time of 900 s are shown in Fig. 6A,B, respectively. With decreasing the potential from 0.5 V to 0.1 V, the value of peak current i_P_ increases along with decreasing of the value of ΔE_P_ for both the BFTO and the SFTO substrates. Also at each applying potential, a noticeable increment in i_P_ and a decrement in $\Delta$E_P_ are seen for the SFTO relative to the BFTO one. From the EIS results, it is seen that with decreasing the deposition potential from 0.5 V to 0.1 V, a considerable decrease in R_et_ value is observed for both the pre-seeded and the bare electrodes (Fig. 6B). It is noticeable that an extreme reduction of R_et_ for the SFTO electrodes relative to the BFTO ones is observed for all positive electrodeposition potentials. Therefore, for the positive potentials, seeding modification results in significant boosting of the electrochemical performance of the electrodes after the electrodeposition of AuNSs.

On the contrary to the positive potentials, there is no noticeable difference between the electrochemical performances of AuNSs deposited on the SFTO and the BFTO electrodes for negative applying potentials, as shown in Fig. 6C,D. Although in this case, the performance of the SFTO is still better. The lack of a significant difference between the two electrodes for the negative potentials may be related to their surface morphologies and the similarly of their surface coverage (see Fig. 3). Comparing the results obtained for the negative and the positive potentials demonstrates that the AuNSs deposited on the SFTO at 0.1 V has the best electrochemical performance in all examined deposition potentials.

The effect of deposition time on electrochemical characteristics of AuNSs for the applied potential of 0.1 V, is shown in Fig. 6(E,F). By increasing the deposition time from 15 min to 60 min, no significant changes in electrochemical performance of AuNSs for the SFTO is observed. While, for the BFTO, the electrochemical performance improves with the deposition time. It is noticeable that the AuNSs deposited on the SFTO for 900 s has a better electrochemical performance in comparison to the 3600 s deposition on the BFTO. Also comparing the results of Figs. S5 and 6E,F reveals that the SFTO with no AuNSs deposition has better electrochemical performances than the BFTO even with AuNSs deposited on it at 0.1 V during 30 min. Based on the results of Fig. 6, the AuNSs on SFTO at 0.1 V for 15 min is the superior electrode for supplementary sensing experiments in this work.

The difference in the i_P_ values of AuNSs deposited on the SFTO compared to the BFTO suggests an improvement of electroactive surface area (ESA) of the AuNSs. The ESA is calculated using the well-known equation of Randles–Sevcik equation^55^:

2 $${I}_{p}=2.69\times {10}^{5}{n}^{3/2}A{C}_{0}{D}^{1/2}{v}^{1/2}$$

with I_p_ the peak current (A), *n* the number of electrons transferred in the reaction, A the electroactive surface area (cm^2^), D the diffusion coefficient (cm^2^ s^−1^), C_0_ the initial bulk concentration of electroactive species (mol cm^−3^), and *v* the scan rate (Vs^−1^). Figure S6 represents the CV curves of AuNSs deposited on the SFTO and SFTO at 0.1 V for 900 s at different scan rates. Based on the slope of I_p_ versus *v*^1/2^ with linear regression equation, the ESA of the AuNSs deposited on the SFTO and the BFTO are obtained as 0.074 and 0.147 cm^2^, respectively. These results indicate significant boosting of the accessible surface area by the seeding modification of the substrate.

## Electrooxidation of glucose

**Figure 7:**
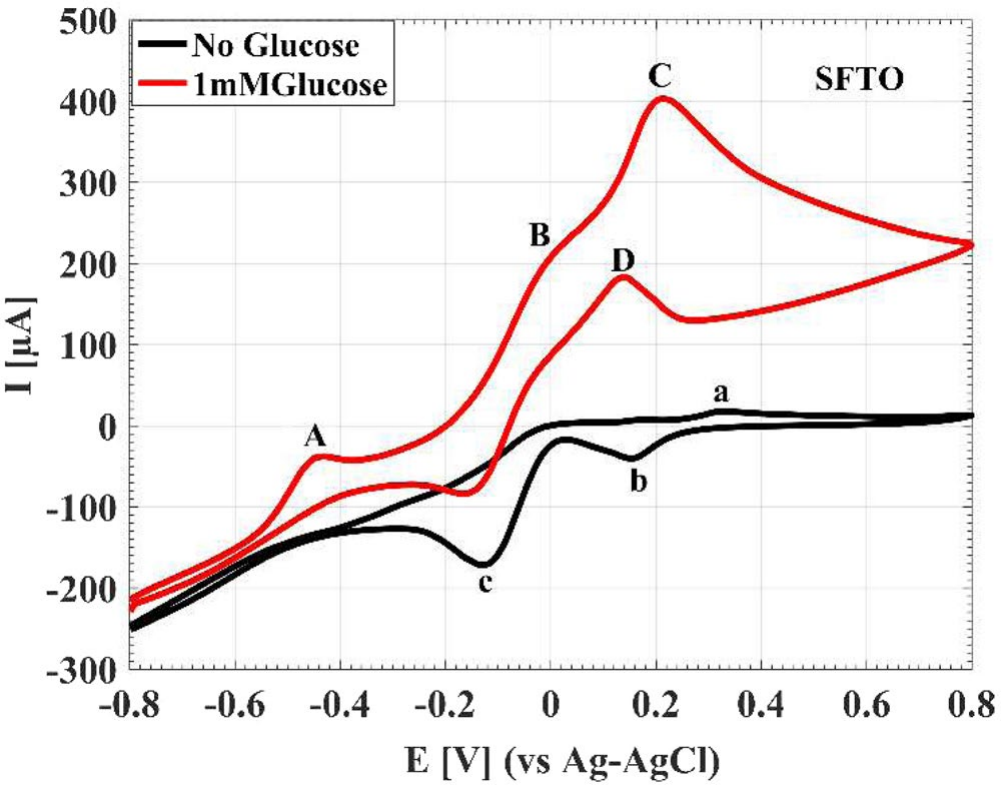
Current versus potential profiles of the superior electrode in the absence and presence of 1 mM glucose in NaOH solution (0.1 M). Scan rate: 20 mV s^−1^.

The electrocatalytic activity of the superior electrode toward glucose oxidation was investigated by measuring CV curves in absence and presence of 1 mM glucose in NaOH electrolyte (0.1 M) at the scan rate of 20 mV s^−1^, as shown in Fig. 7. In the absence of glucose, the CV curve shows three substantial peaks. In the forward scan, the anodic peak at 0.33 V correspondes to the oxidation of gold. In the backward scan, the first cathodic peak relates to the reduction of gold oxides at 0.15 V and second cathodic peak corresponds to desorption of hydroxide layer at −0.13 V. After the addition of 1 mM glucose to the electrolyte, several CV peaks associating to the glucose oxidization appear. During the positive potential scan, a rise in the anodic current is observed at the onset potential of −0.80 V in addition to the three main anodic peaks at −0.45 V (A), −0.06 V (B) and 0.21 V (C). The first peak (A) imputes to the partial oxidation of glucose. The peak at −0.06 V (B) corresponds to subsequent oxidation of the gluconolactone^56^. At more positive potentials, the density of AuOH sites increases on the substrate surface, which catalyzes oxidation of gluconolactone, and provides new active sites for the direct oxidation of glucose leading to the peak at 0.21 V (C). The decrease in the current after the third peak is induced due to the oxidation of gold surfaces, leading to the decline in the density of AuOH sites and inhibiting the oxidation of glucose. In the backward scan, due to the reduction of the gold oxide, sufficient active sites will be accessible for the direct oxidation of glucose, making an cathodic peak at 0.13 V (D). Along with the potential moving to more negative values, the partial oxidation of glucose at the sites of AuNSs occurs again that results in the rise of the gluconolactone quantity on the surface and consequently increasing the current in the potential range of −0.16 V to −0.4 V^57^.

A comparison between the electrocatalytic activity of AuNSs deposited on the SFTO with the BFTO is shown in Fig. S7. In the absence of glucose (Fig. S7A), the CV current peaks of the AuNSs on the SFTO are considerably higher than the BFTO indicating high surface activity of the seed modified electrode. After the addition of 1 mM glucose, as shown in the Fig. S7B, the BFTO displays a peak of partial oxidation of glucose at about −0.28 V whereas the partial oxidation peak of the SFTO occurs at considerably lower anodic potential at −0.45 V. At more positive potentials, the seed modified electrode shows higher rates of reaction in comparison with the bare one so that at the direct oxidation potential of 0.21 V, the maximum current is 3.3-fold higher than that of the BFTO. It can be seen, in backward scan that the seed modified electrode exhibits higher rates of reaction than that of the bare one. The remarkable decrease in the partial oxidation potential, the higher reaction rates and the 3.3-fold higher current for AuNSs on the SFTO in comparison with the BFTO demonstrate the significant improvement of glucose electrooxidation occurring by the seeding modification.

**Figure 8:**
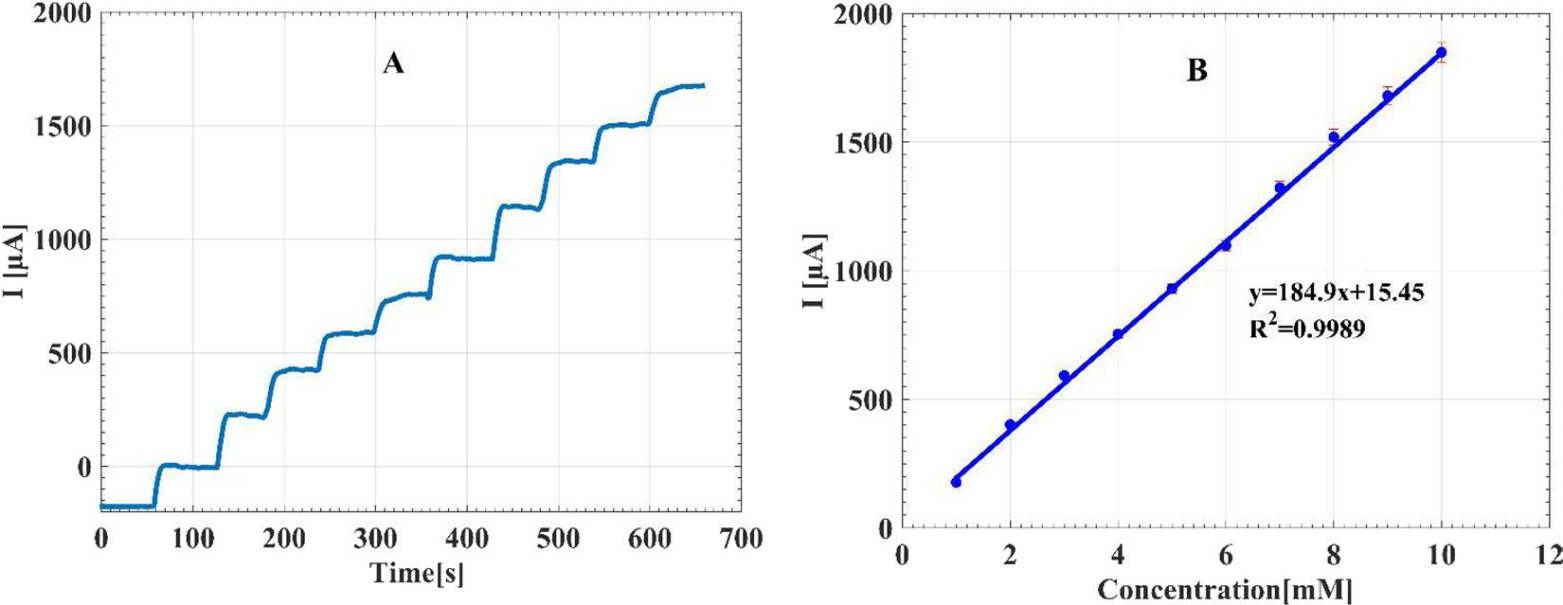
(**A**) The amperometric responses of the AuNSs to the successive addition of glucose with the concentrations from 1 mM to 10 mM in NaOH solution (0.1 M) at the constant potential of −0.45 V. (**B**) The corresponding calibration curve of the sensor for various glucose consentrations.

For the electrochemical sensing applications, amperometric detection is usually employed. For amperometric detection, it is essential to find a proper detection potential to achieve the highest sensitivity and selectivity responses. As mentioned above, all corresponding peak potentials can be utilized to investigate the catalytic activity of the electrode. Since the detection at lower potentials such as −0.45 V eliminates easily oxidizable species such as ascorbic acid (AA) and uric acid (UA), we selected the potential of −0.45 V for the amperometric detection of glucose^56,58^. Figure 8 shows the amperometric response and the calibration curve of the AuNSs on the SFTO at the constant potential of −0.45 V for successive additions of 1 mM glucose in the NaOH electrolyte (0.1 M). From the calibration curve, a linear range to the glucose detection is obtained in the concentration range from 1–10 mM with a sensitivity of 184.9 µA mM^−1^ cm^−2^, achieved from the slope in the linear range. The performance of this sensor is compared to some of the other published non-enzymatic sensors based on AuNSs embeded on the surface, as listed in Table S1. Based on the desirable sensitivity, limit of detection, and linear response range that a typical non-enzymatic glucose sensor should have, the sensor of the current work can fulfill all required specifications for the practical applications. The remarkable properties of the current sensor is related to high surface area and excellent electrical conductivity of the electrode, which leads to fast and direct electron transfer during the electrooxidation of glucose.

The selectivity of the sensor was studeied in presence of common interference species such as UA, and AA in addition to other sugars including lactose, sucrose, mannose, and fructose, in physiological levels^59,60^. Addition of 0.02 mM UA, 0.1 mM AA, and also other sugars to a 1 mM glucose solution lead to only 0.2–4% increase in the current and does not interfere with the glucose detection, as shown in Fig. S8A. In addition, the selectivity of the sensor in the presence of 0.1 M NaCl, 0.1 M MgCl_2_, 0.01 mM tyrosine (Tyr), 0.03 mM phenylalanine (Phe) and 0.1 mM glutamine (Gln) is shown in Fig. S8B.

The operational stability was studied by measuring the CV curves for several cycles. The current response of the electrode to 20 cycles in the presence of 1 mM glucose is displayed in Fig. S8C. It is seen that from the third cycle to the next ones, the current response remains stable during the electrochemical measurements. The storage stability is also examined by amperometric determination of glucose at −0.45 V for periods of 5 days. The results show only 5% decrease in current response respect to its initial response after one-month (Fig. S8D), which indicates that the electrode is stable and can be suitably used in all practical measurements of glucose.

The practical utility of the sensor was studied by glucose measuring for 3 human serum samples, and the results were compared with the reference values obtained from clinical measurements in Shariati hospital of Tehran (Iran). As shown in Table S2, it is seen that the obtained results from sensor exhibit an excellent agreement with the clinical measurements with less than 3.2% deviation. This indicates that the proposed sensor of this work can well be employed for practical testing with beneficial accuracy. Also, the recovered test has been performed for the sensor, and the results have been shown in Table S2 too. The selected serum samples were injected with known glucose concentrations of 0.5 mM. It was found that the recovery range is between 98–103% for the examined human serums. In addition, the relative standard deviation (RSD) of the glucose measurements of the human samples were found between 2.37–3.2.

## Conclusion

In this work a seeding modification approach has been applied to enhance electrochemical performance of AuNSs. A template-less and surfactant free electrodeposition method has been used to direct growth of AuNSs on the BFTO and SFTO. The electrochemical measurements reveal noticeable enhancement of the active surface area and charge transfer performance of AuNSs due to the pre-seeding of the substrates. The influences of electrodeposition potential and time on the morphology and electrochemical properties of the fabricated AuNSs have been investigated. Based on the electrochemical results, the AuNSs deposited at 0.1 V for 900 s on the SFTO was selected as the superior electrode for glucose detection. The results show excellent electrocatalytic activity of this electrode toward glucose oxidation with presenting a high sensitivity of 184.9 µA mM^−1^ cm^−2^ and a low limit of detection of 0.32 µM.The electrode presents remarkable anti-interference characteristics, operational and storage stabilities.

## Methods

### Materials and chemicals

Au metallic pellets were purchased from Kurt J. Lesker. Fluorine-doped tin oxide (FTO) substrates obtained from Sharifsolar company, with a thickness of 3.3 mm and surface resistance of 20 Ωsq^−1^. Chloroauric acid (HAuCl_4_), potassium ferricyanide [K_3_Fe (CN)_6_], potassium ferrocyanide [K_4_Fe (CN)_6_], Glucose, NaOH, and other reagents were bought from Merck Company. All chemical materials were of analytical grade and used without further purification. All solutions were prepared with Milli-Q water (18.2 MΏcm).

### Apparatus

Typical electrochemical measurements and depositions were carried out with an Origalys (ElectroChem SAS, France) potentiostat/galvanostat system. The conventional three-electrode cell comprising a modified working electrode, a platinum foil auxiliary electrode, and an Ag-AgCl reference electrode, was used for all electrochemical experiments. The surface morphology of the fabricated electrodes was characterized with field emission scanning electron microscopy (FESEM), FEI Nova NanoSEM 450 model. The surface topographies of the fabricated electrodes were acquired by atom force microscopy (AFM, Nanosurf Flex, Swiss). X-ray diffraction (XRD) pattern were collected on the X’Pert PRO MPD system equipped with Cu-Kα radiation. Additionally, all the experimental measurements were carried out at the room temperature.

### Substrate preparation

Prior to use, FTO substrates, which were selected as the bare electrodes, were cut to 20 × 8 mm^2^ pieces. After that, they were rinsed with double distilled water, and then ultrasonicated in double distilled water, acetone and ethanol for 15 min successively. Finally, they were dried inside an oven to be ready for deposition.

### Seeding modification of the FTO substrates by gold nanoparticles

A thin film of Au with nominal thickness of 5 nm was deposited on the FTO substrate by a custom-designed thermal evaporation system. During evaporation, the pressure of the chamber and deposition rate were adjusted at 5 × 10^−6^ Torr and 0.01 nm s^−1^, respectively. The film thickness was measured by a quartz crystal microbalance (QCM). Then, a thermal annealing treatment has been carried out for the deposited Au films at the temperature 500 °C for 10 h under the air atmosphere. The heating rate was fixed at 15 °C min^−1^, and after the annealing, the temperature was decreased to the room temperature. Finally, the electrodes modified by AuNPs are obtained which is called as “pre-seeded” substrates.

### Electrochemical growth of AuNSs

For this purpose, a standard three-electrode cell was employed for direct electrochemical deposition. First, the precursor electrolyte solution was provided by HAuCl_4_ (0.5 mM). Then, the pre-seeded FTO substrates or the bare ones were dipped in the electrolyte solution, as the working electrodes. A platinum foil and an Ag-AgCl electrode were utilized as the counter and the reference electrodes, respectively. Subsequently, the electrochemical growth of AuNSs was executed under potentiostatic technique at the room temperature. After that, the modified electrode was rinsed three times with double distilled water and withered with a flow of the air.

## Supplementary information

Supplementary Information.
