# Supplementary material for: Seed-mediated Electrochemically Developed Au Nanostructures with Boosted Sensing Properties: An Implication for Non-enzymatic Glucose Detection
Source: Sci Rep. 2020 Apr 29;10:7232. doi: 10.1038/s41598-020-64082-5 (PMC7190711; doi:10.1038/s41598-020-64082-5)
Supplement: Supplementary file 1 — Supplementary Information. [file 41598_2020_64082_MOESM1_ESM.docx]

Seed-mediated Electrochemically Developed Au Nanostructures with Boosted Sensing Properties: An Implication for Non-enzymatic Glucose Detection

*Hossein Siampour^1^, Sara Abbasian^2^, Ahmad Moshaii^1*^, Kobra Omidfar^3^, Mosslim Sedghi^4^, Hossein Naderi-Manesh^4^*

^1^Department of Physics, Tarbiat Modares University, Tehran, P.O Box 14115-175, Iran

^2^School of Physics, Institute for Research in Fundamental Sciences (IPM), P.O. Box 19395-5531, Tehran, Iran

^3^Biosensor Research Center, Endocrinology and Metabolism Molecular-Cellular Sciences Institute, Tehran University of Medical Science, Tehran, Iran

^4^Department of Nanobiotechnology/Biophysics, Faculty of Biological Sciences, Tarbiat Modares University, Tehran 14115-154, Iran

E-mail: [moshaii@modares.ac.ir](mailto:moshaii@modares.ac.ir)

| 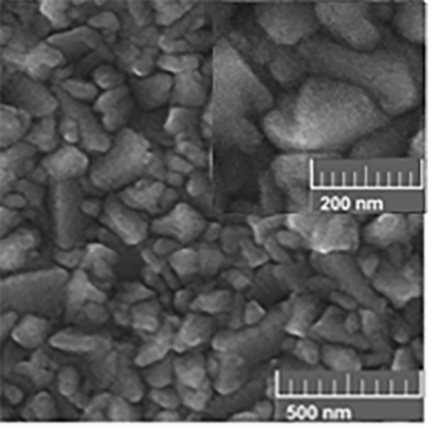  **A** |   **B** | 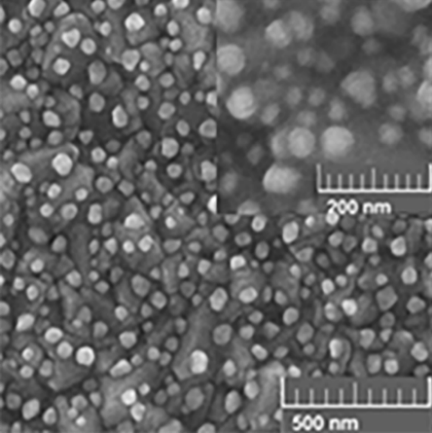  **C** |
| --- | --- | --- |

Figure S1. FESEM images of (A) the bare electrode, (B) the 5 nm gold film deposited on the bare electrode and (C) the Au-NPs after the thermal treatment.


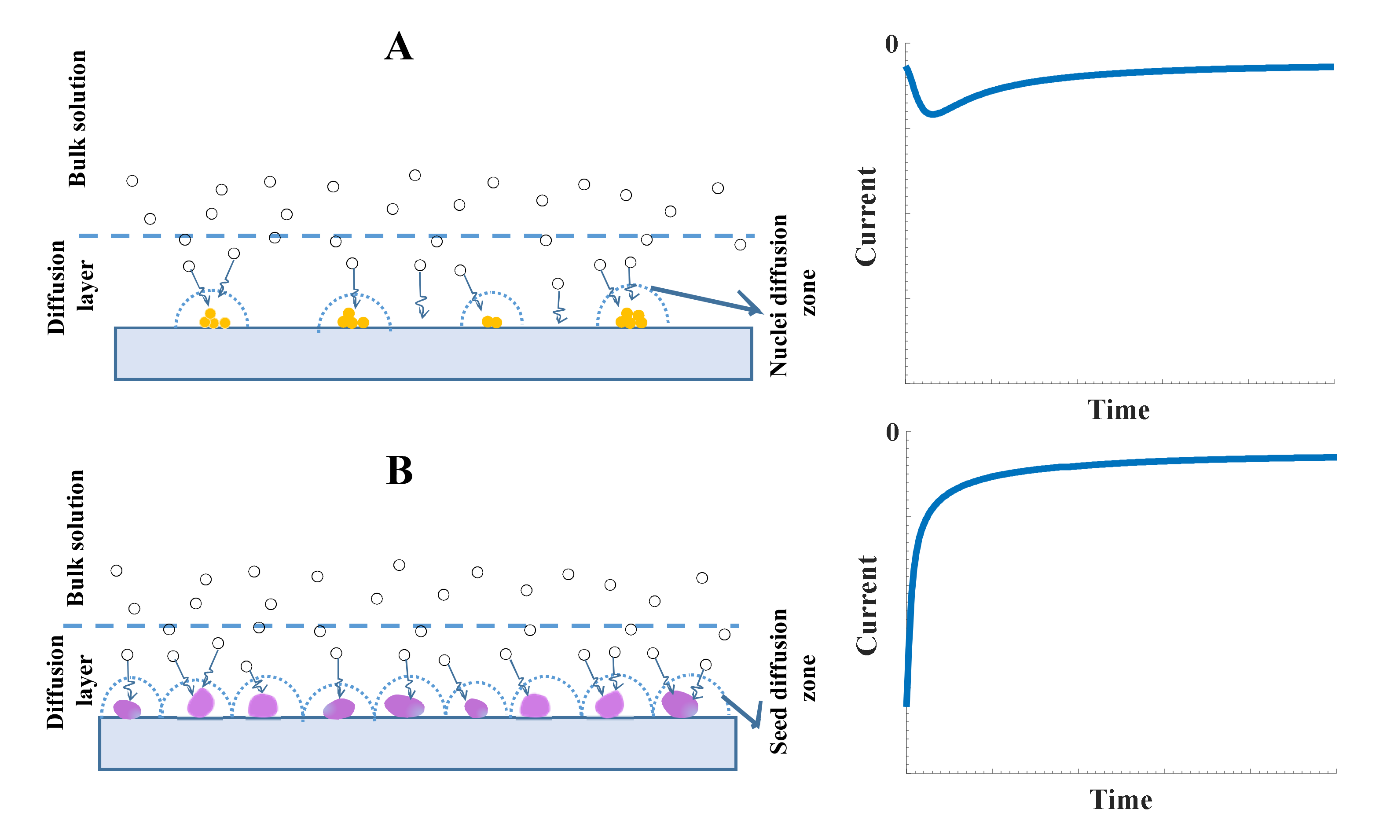


Figure S2. Schematic illustration of the process of fabricating AuNSs and their electrodeposition amperometric curves for the bare (A) and the pre-seeded (B) electrodes.

Figure S3 shows the XRD patterns of the bare FTO (BFTO), the pre-seeded FTO (SFTO), and the AuNSs electrodeposited at the applied potential of 0.1 V for 900 s on SFTO (SFTO:15 min). The peaks obtained at 2θ= 27.1°,34.06°, 39°, 52.8°, 55.1°, 62°, 65.8°, 72°,79.15°, and 84.5° are the characteristic peaks of the BFTO. In the case of SFTO, two diffraction peaks of 38.4° and 44.9° are corresponding to the (111) and (200) planes of Au demonstrating the formation of seeding Au nanoparticles on the substrate. For electrodeposited AuNS, the diffraction peaks at 38.4°,44.9°, 64.9°,78° and 81.9° are corresponding to the (111), (200), (220), (311) and (222) planes and indicate formation of a FCC crystalline Au structure. The disappearance of the diffraction peaks of FTO for the lather case, shows enough loading of Au on the substrate.


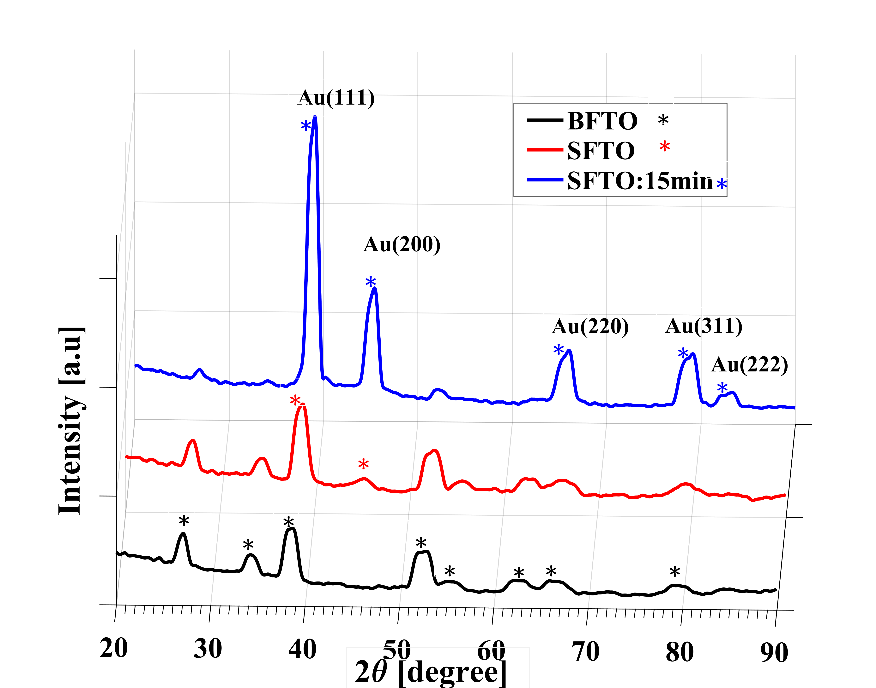


Figure S3. XRD patterns of the bare electrode (BFTO), the pre-seeded electrode (SFTO), and the electrodeposited Au nanostructures on the SFTO at an applied potential of 0.1 V for 900 s (SFTO:15 min).

The AFM images of BFTO, SFTO and SFTO:15 min and their corresponding height profiles are shown in Fig. S4. The black and the blue arrows are relating to seeding Au nanoparticles and the electrodeposited AuNSs, respectively. The corresponding heights of the points indicated on the AFM images are highlighted in the height profiles (yellow). Increasing the roughness in the height profile after the electrochemical growth confirms the growing of AuNSs on the seeding nanoparticles. The result of AFM well agrees with SEM images in which the growing of AuNSs on the seeding nanoparticles is noticeable.


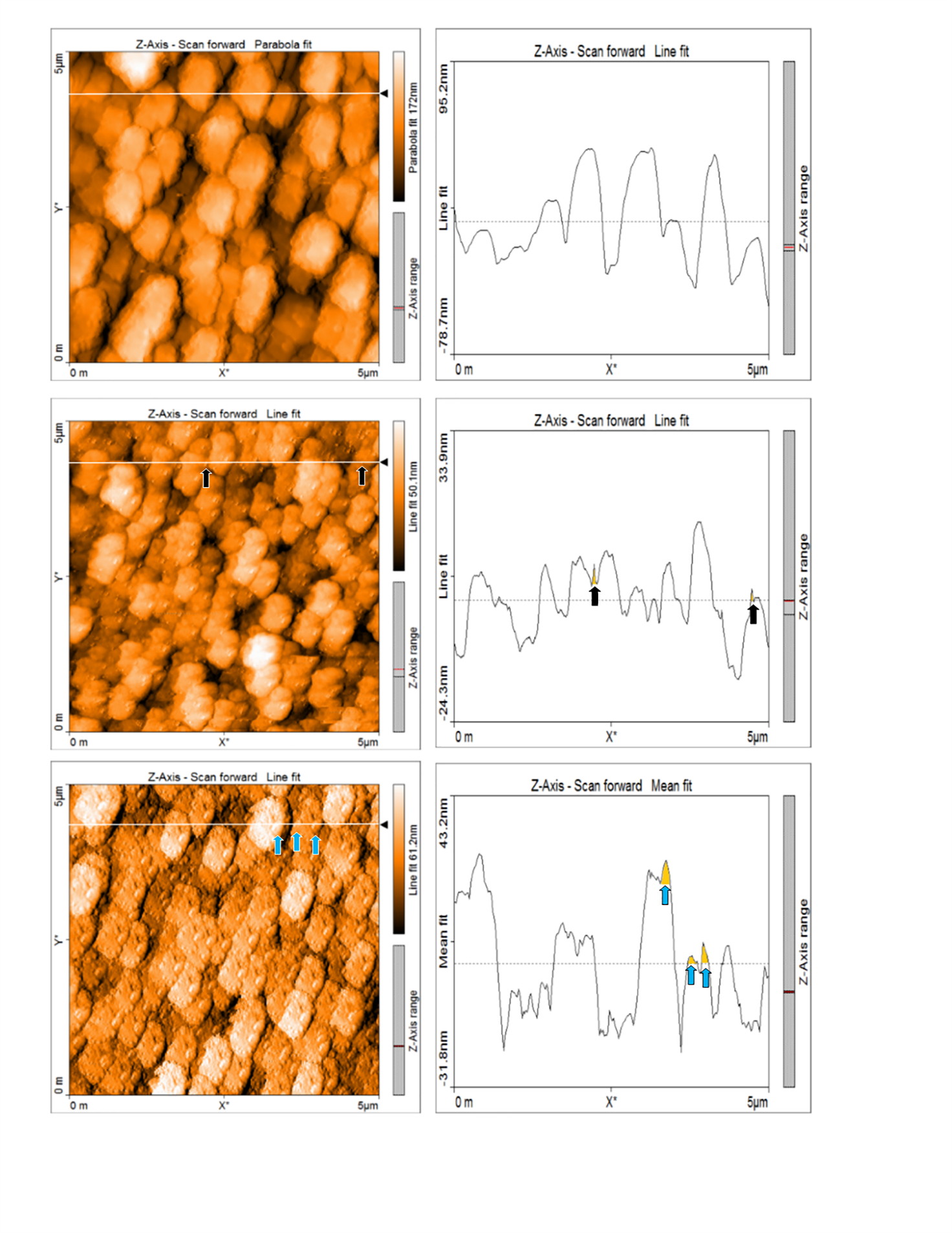


C

B

A

**Figure S4**. AFM images of the BFTO (A), the SFTO (B), and the SFTO: 15 min (C), and their corresponding height profile.

| 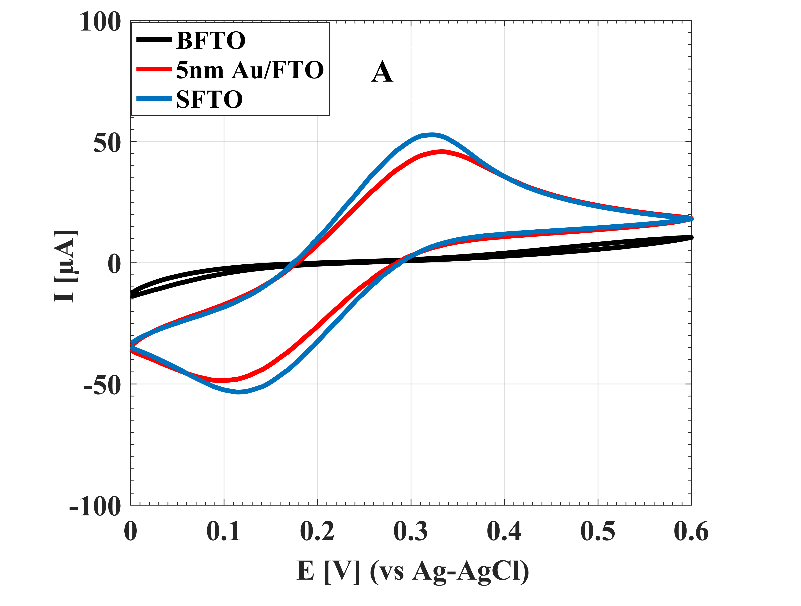 | 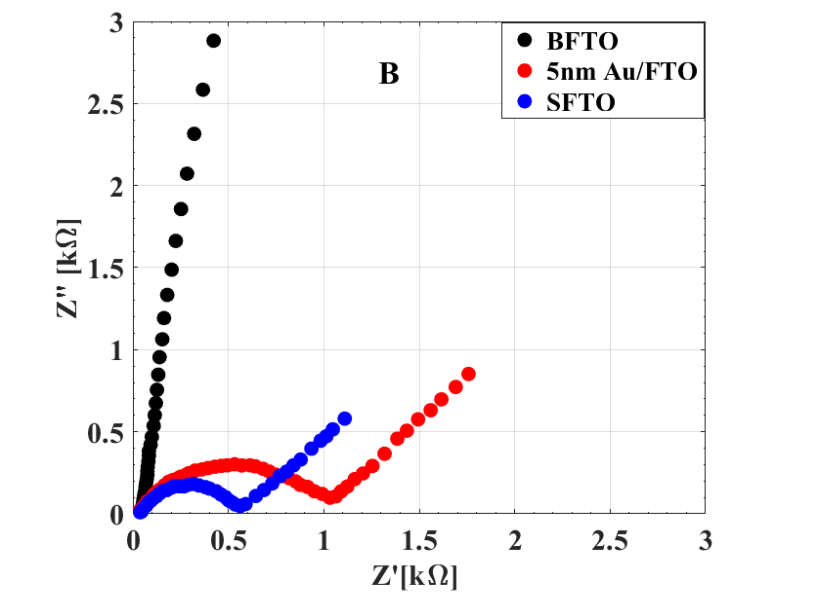 |
| --- | --- |

Figure S5. Cyclic voltammograms (A) and Nyquist plots (B) of the BFTO, 5 nm Au/FTO and the SFTO in KCl solution (0.1 M) containing redox couple of [Fe (CN)_6_]^3-/4^ (2.5 mM).

| 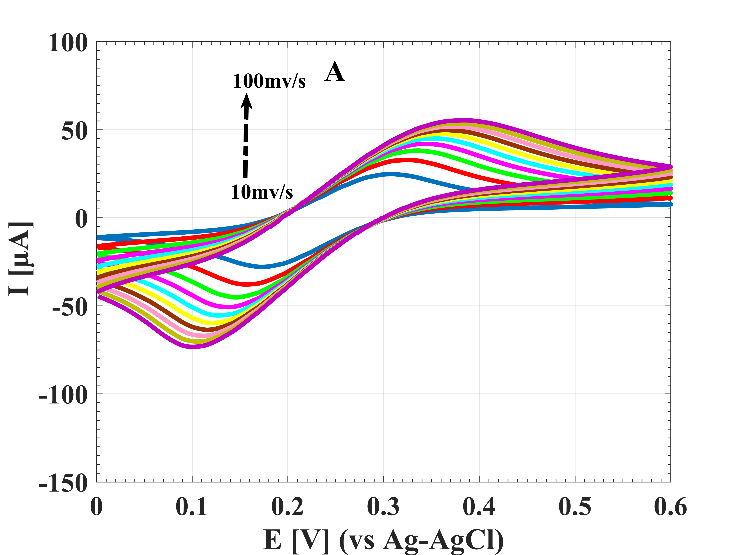 | 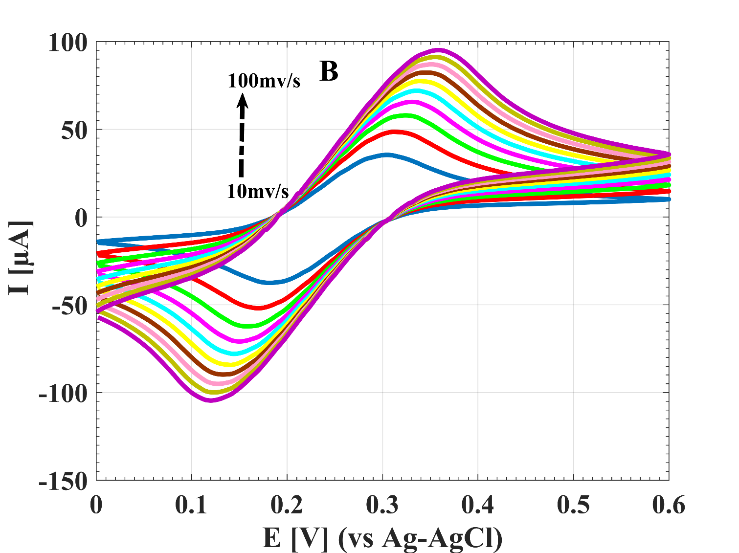 |
| --- | --- |
| 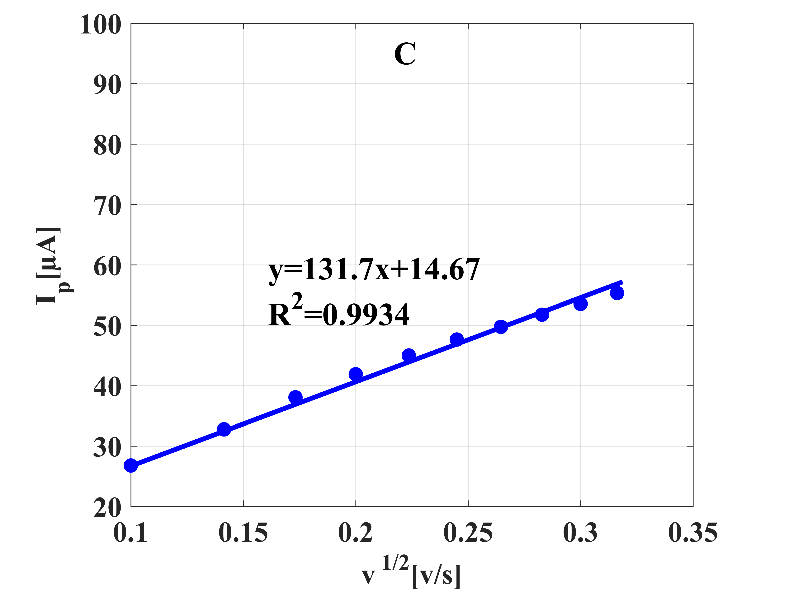 | 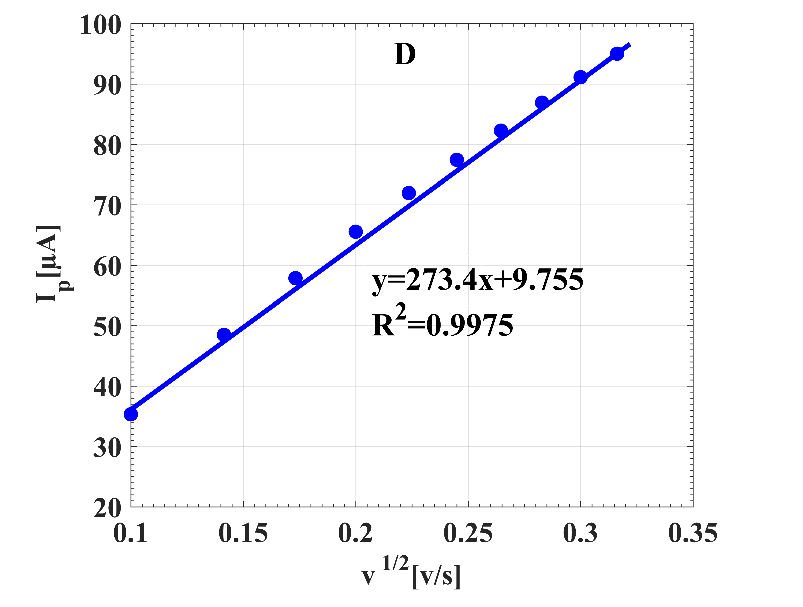 |

Figure S6. The CV curves of the BFTO:15 min (A) and SFTO:15 min (B), in KCl solution(0.1 M) containing redox couple of [Fe (CN)_6_]^3-/4^ (2.5 mM) over the potential range of 0V to +0.6 V at various scan rates ranging from 10-100 mV s^-1^. Graphes C and D display the calibration plot of peak current vs. V^1/2^ for the BFTO:15 min and the SFTO:15 min, respectively.

| 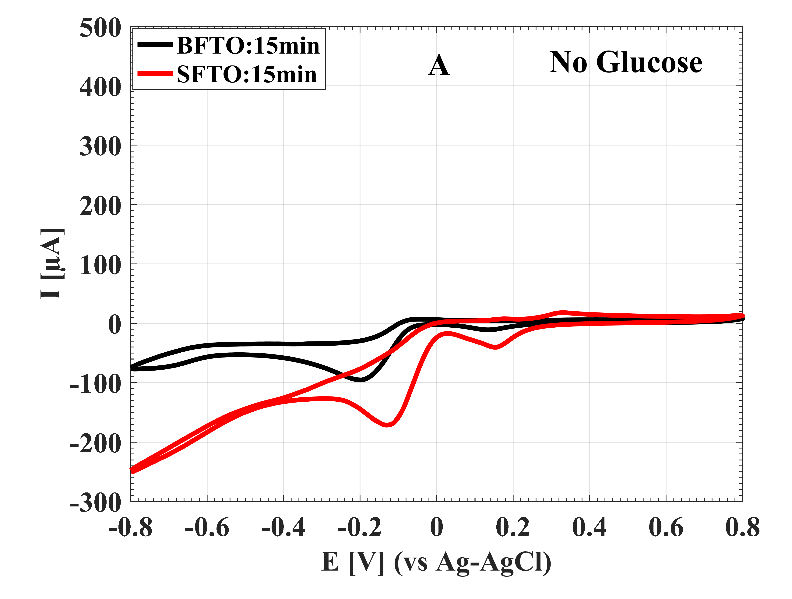 | 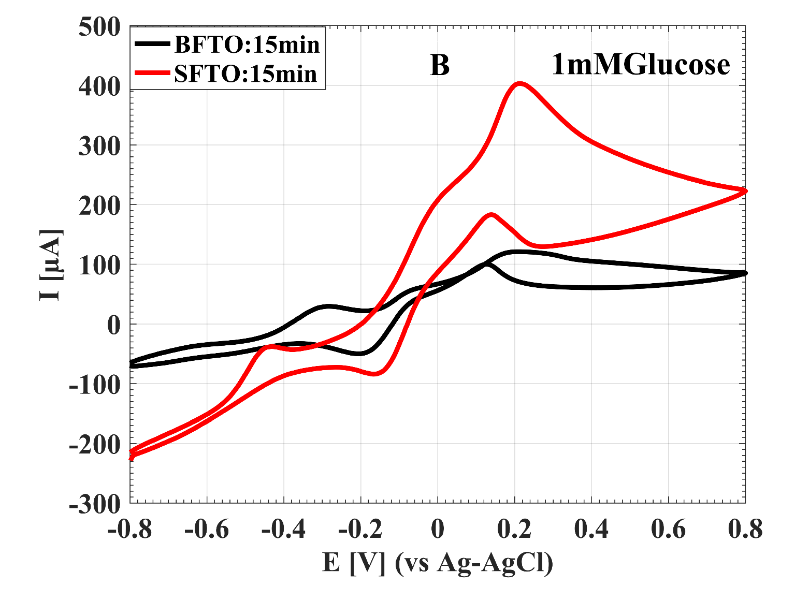 |
| --- | --- |

Figure S7. The CV curves of the BFTO:15min and SFTO:15 min in the absence (A) and the presence (B) of 1 mM glucose in NaOH solution (0.1 M) with scan rate: 20 mV s^−1^.

| **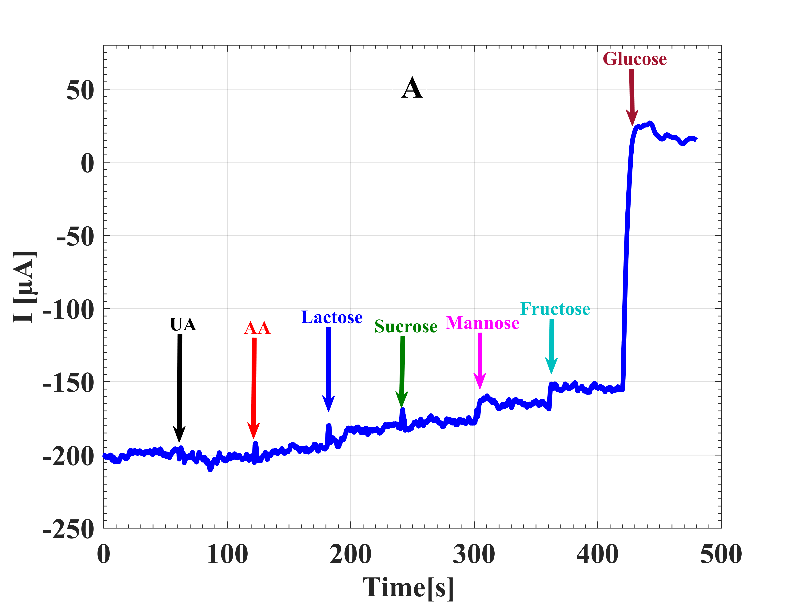** | 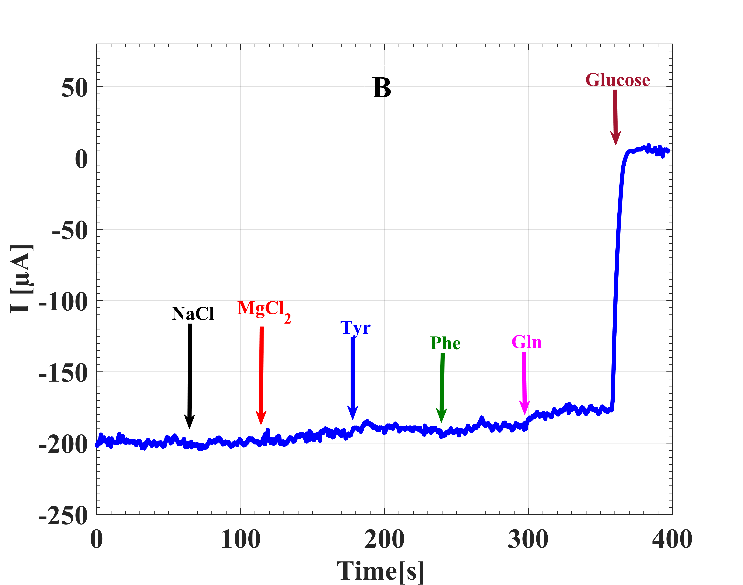 |
| --- | --- |
| 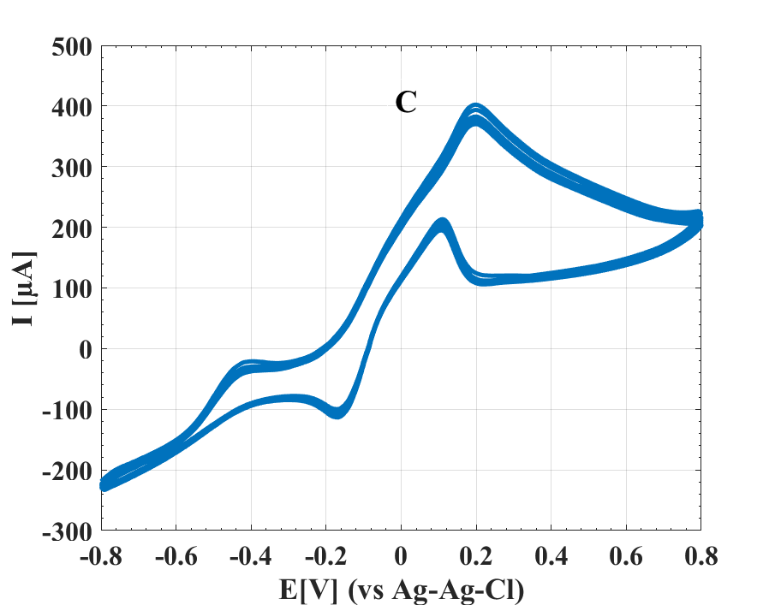 | 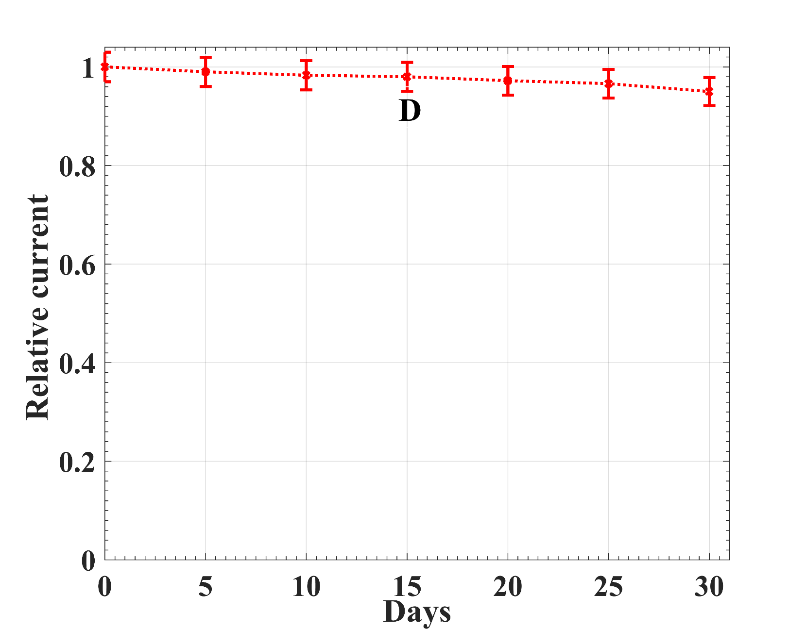 |

**Figure S8**. (A): Interference test of the sensor with 0.02 mM UA, 0.1 mM AA and 0.1 mM lactose, sucrose, mannose, fructose, as well as 1 mM glucose at the potential of -0.45 V. (B): Interference test of the sensor in the presence of 0.1 M NaCl, 0.1 M MgCl_2_, 0.01 mM tyrosine (Tyr), 0.03 mM phenylalanine (Phe) and 0.1 mM glutamine (Gln) as well as 1 mM glucose at the potential of -0.45 V. (C): The CV curves of the sensor in the present of 1 mM glucose for 20 cycles. (D): The change of current of sesnor against to 1 mM glucose for different periods of 5 days.

**Table S1**. Comparison of the performance of various Au based electrochemical non-enzymatic glucose sensors with the current work.

| Electrode | Operational potential(V) | Sensitivity(µA mM^−1^ cm^−2^) | Linear range(mM) | Detection limit(µM) | HAuCl_4_ solution(mM) | Reference |
| --- | --- | --- | --- | --- | --- | --- |
| mesoporous Au film | 0.2 | 291.6 | 0.01-10 | 4.13 | 40 | ^1^ |
| Au disk / MWCNT-CG | 0.2 | 27.7 | 0.001-1 | 0.5 | 114.78 | ^2^ |
| Au-cluster/FTO | 0.1 | 10.65 | 0.01-10 | 2 | 10 | ^3^ |
| AuNPs array/ITO | 0.15 | 23 | 0-11 | 5 | 0.1 | ^4^ |
| Au-nanotube array/GCE | 0.25 | 44.2 | 0.005-16.4 | 2.1 | 35.31 | ^5^ |
| Gelatin -templated Au NSs | 0 | 73.6 | 0.001-0.5 | 1.5 | 7.8 | ^6^ |
| Hole-possess spherical AuNSs/FTO | -0.45 | 184.9 | 1-10 | 0.32 | 0.5 | This work |

Table S2. Determination of glucose concentrations in blood serum samples

| Samples | Clinical results (mM) | This sensor  (mM) | RSD  (n = 3)(%) | Difference (%) | Glucose added  (mM) | Recovery test  (%) |
| --- | --- | --- | --- | --- | --- | --- |
| Serum 1 | 4.38 | 4.24 | 2.37 | 3.2 | 0.5 | 98 |
| Serum 2 | 4.13 | 4.05 | 2.41 | 1.9 | 0.5 | 101 |
| Serum 3 | 6.47 | 6.36 | 3.2 | 1.7 | 0.5 | 103 |

**References**

1. Nugraha, A. S. *et al.* Block‐Copolymer‐Assisted Electrochemical Synthesis of Mesoporous Gold Electrodes: Towards a Non‐Enzymatic Glucose Sensor. *ChemElectroChem* **4,** 2571–2576 (2017).

2. Kangkamano, T., Numnuam, A., Limbut, W., Kanatharana, P. & Thavarungkul, P. Chitosan cryogel with embedded gold nanoparticles decorated multiwalled carbon nanotubes modified electrode for highly sensitive flow based non-enzymatic glucose sensor. *Sensors Actuators B Chem.* **246,** 854–863 (2017).

3. Han, L. *et al.* Porous gold cluster film prepared from Au@ BSA microspheres for electrochemical nonenzymatic glucose sensor. *Electrochim. Acta* **138,** 109–114 (2014).

4. Wang, J., Cao, X., Wang, X., Yang, S. & Wang, R. Electrochemical oxidation and determination of glucose in alkaline media based on Au (111)-like nanoparticle array on indium tin oxide electrode. *Electrochim. Acta* **138,** 174–186 (2014).

5. Tian, T., Dong, J. & Xu, J. Direct electrodeposition of highly ordered gold nanotube arrays for use in non-enzymatic amperometric sensing of glucose. *Microchim. Acta* **183,** 1925–1932 (2016).

6. Juſík, T. *et al.* Nanostructured gold deposited in gelatin template applied for electrochemical assay of glucose in serum. *Electrochim. Acta* **188,** 277–285 (2016).
